# Using integrated problem- and lecture-based learning teaching modes for imaging diagnosis education

**DOI:** 10.1186/s12909-018-1303-2

**Published:** 2018-08-02

**Authors:** Jun-Yan Yue, Jie Chen, Wen-Guang Dou, Chang-Hua Liang, Qing-Wu Wu, Yi-Yong Ma, Zhi-Ping Zhu, Mei-Xia Li, Yan-Long Hu

**Affiliations:** Department of Radiology, The First Affiliated Hospital of Xinxiang Medical University, 88 Jiankang Road, Weihui City, Henan Province China

**Keywords:** Problem-based learning, Lecture-based learning, Imaging diagnosis, Teaching mode, Survey

## Abstract

**Background:**

There are two parts included in traditional imaging diagnosis teaching: theoretical lessons and experimental lessons. Most of the time, the experimental lesson is a review of the theoretical lesson. The teacher is the centre of the course and students are passive learners. Thus, in this study we included the patient problem of the imaging centre in our imaging diagnosis education. The traditional theoretical lessen was used to discuss prior knowledge, the discussion and analysis of patient problems was arranged under class, and the experimental lesson was used to synthesize and test the newly acquired information. The aim of this study is to determine whether or not integration of problem- and lecture-based learning teaching modes in imaging diagnosis education was associated with a good teaching effect. Forty-six of sixty students (76.7%) like integrated problem- and lecture-based learning teaching mode and 53 of 60 students (88.3%) think that integrated problem- and lecture-based learning teaching mode can make their ability of self-study be improved.

**Methods:**

Sixty students participated in a prospective study with a two-phase cross-over design. All of the students were divided into 2 groups of 30 each. In the first term, the first group participated in an integration of the problem- and lecture-based learning teaching mode, whereas students in the second group underwent the lecture-based learning teaching mode alone. During the second term, the teaching modes were exchanged between the two groups. A close-exam and survey were used to evaluate the teaching effect, and the data were analysed means of analysis of variance with a two-phase cross-over design and a χ^2^ test with a 2-tailed *α* of 0.05.

**Results:**

There was a statistically significant difference in the test scores between the integration of the problem- and lecture-based learning teaching mode and the lecture-based learning teaching mode alone (*P* < 0.05). The integration of problem- and lecture-based learning teaching mode was well-appraised.

**Conclusion:**

Integration of the problem- and lecture-based learning teaching modes in teaching imaging diagnosis education resulted in a good teaching effect.

## Background

Problem-based learning (PBL) was originally developed by the faculty of the Health Sciences of McMaster University in 1965 [1]. PBL is an instructional method that is thought to provide students with knowledge suitable for problem solving and includes three principles of learning: activation of prior knowledge; elaboration; and encoding specificity. The process of PBL starts with a problem provided by the teacher, then the study group works on the problem, which involves the following seven steps: clarify terms and concepts not readily comprehensible; define the problem; analyse the problem; draw a systematic inventory of the explanations inferred from analysing the problem; formulate learning objectives; collect additional information outside the group; and synthesize and test the newly acquired information [2]. In most cases, a problem consists of a description of a set of phenomena or events that can be perceived in reality [1]. The conception of PBL in medical education presented in the 1980 publication was a culmination of 15 years of research and development, including implementation of the first PBL medical curriculum at McMaster. In recent decades, there has been lively debate among educators about the value of the PBL teaching method in undergraduate medical education [3, 4]. Some research has shown that a PBL curriculum is a potential and promising teaching method that can be widely utilized [5], and other research has shown that PBL is rated highly by students and faculty as a valuable learning methodology that enhanced their knowledge and understanding of patient problems, thus providing a more holistic approach to care [6]. Some have argued that in a few instances PBL students scored lower on basic sciences examinations and viewed themselves as less well prepared in the basic sciences than conventionally-trained counterparts [7], and some argue that practical experiences are more important than the teaching method used to achieve a student’s perceived competence [8].

The discussion and analysis of patient problems is the core of the PBL curriculum, the engine that drives learning, and the arena in which cognitive skills, that are the foundation of clinical reasoning, are developed [4]. In an imaging centre, the patient problems correspond to the imaging diagnosis of the pictures. There are two parts included in traditional imaging diagnosis teaching: theoretical lessons (approximately 80 min); and experimental lessons (approximately 160 min). In the theoretical lessons, teachers talk about the conception, the pathologic, clinical, and imaging findings of the disease, and the differential diagnosis of the disease. In the experimental lessons, teachers provide imaging pictures. In the initial part of the class, teachers discuss the diagnosis based on the pictures and the imaging findings, and in the latter part of the class, the students evaluate the pictures and ask questions. From the course of imaging diagnosis teaching, we can see that there is a lot of repetition in theoretical and experimental lessons. Most of the time, the experimental lesson is a review of the theoretical lesson. The teacher is the centre of the course and students are passive learners. Thus, in this study we included the patient problem of the imaging centre in our imaging diagnosis education. The traditional theoretical lessen was used to discuss prior knowledge, the discussion and analysis of patient problems was arranged under class, and the experimental lesson was used to synthesize and test the newly acquired information.

The aim of this study was to evaluate whether or not integration of the PBL and lecture-based learning (LBL) teaching modes imported into the teaching of imaging diagnosis education gave rise to a good teaching effect.

## Methods

**Fig. 1:**
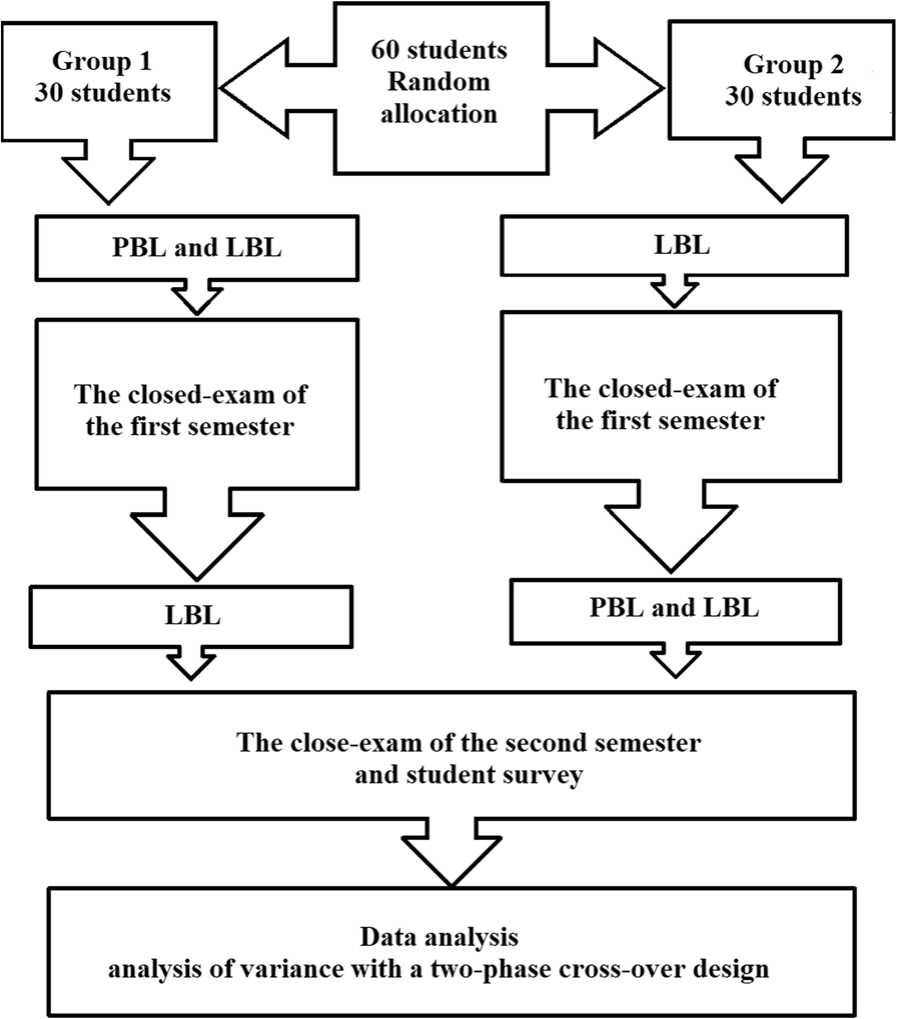
shows the entire process of the research design

The entire process is shown in Fig. 1.

### Participants

During the 2016–2017 academic year, a portion of the Xinxiang Medical University fourth year imageology clerks participated in a pilot study aimed at improving learning and instruction. There were 36 theoretical and experimental lessons each in the first term and 17 lessons in the second term. There were 60 students, all of whom were randomly divided into 2 groups with 30 students in each group. There were no statistical differences in the age, gender, and scores of the entrance exams and school performance between the two groups. During the first term, 30 students in the first group were divided into 10 teams. Every team included 3 team members who participated in the integration of PBL and LBL, whereas students in the second group underwent the LBL teaching mode alone. During the second term, the teaching modes were switched between the two groups. This was a prospective study that was conducted to analyse the usefulness of integrating PBL and the traditional teaching modes. The study was evaluated by the Institutional Review Board of the First affiliated Hospital of Xinxiang Medical University. Informed consent was waived by the Institutional Review Board.

### Methods

**Fig. 2:**
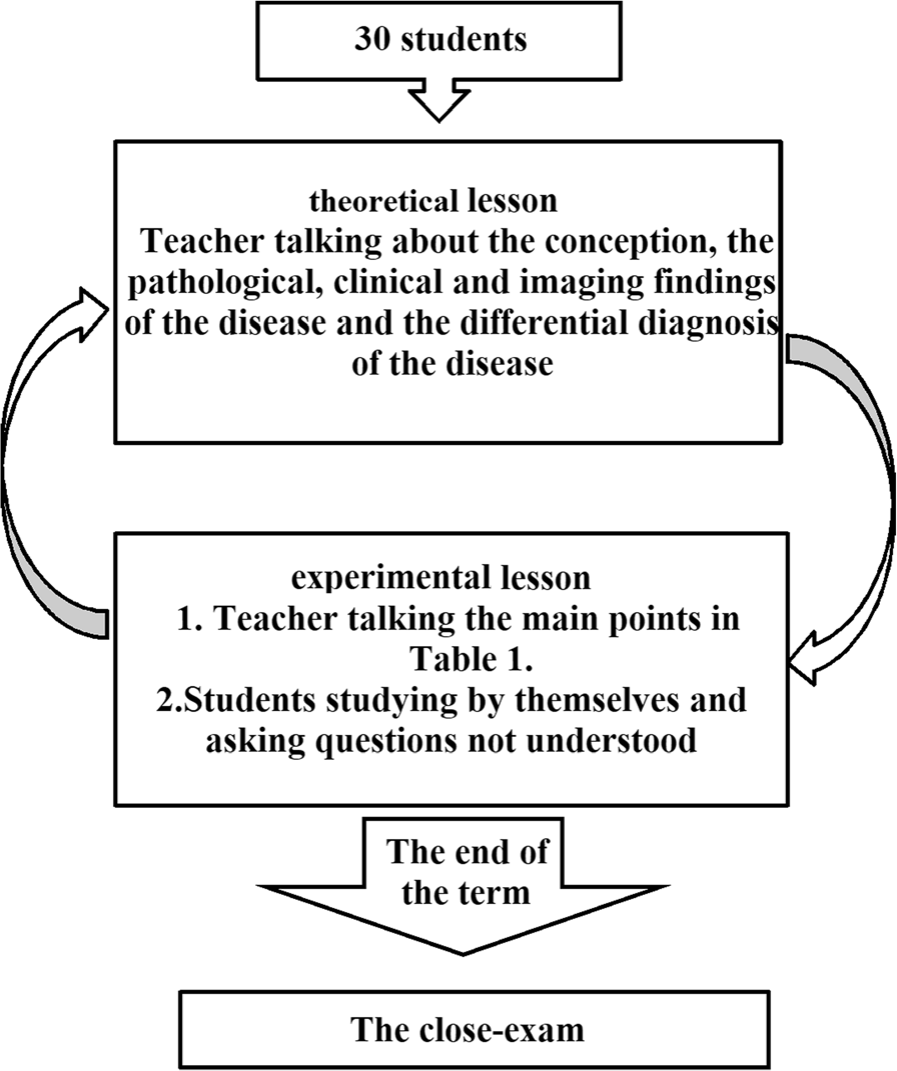
shows the process of LBL

**Fig. 3:**
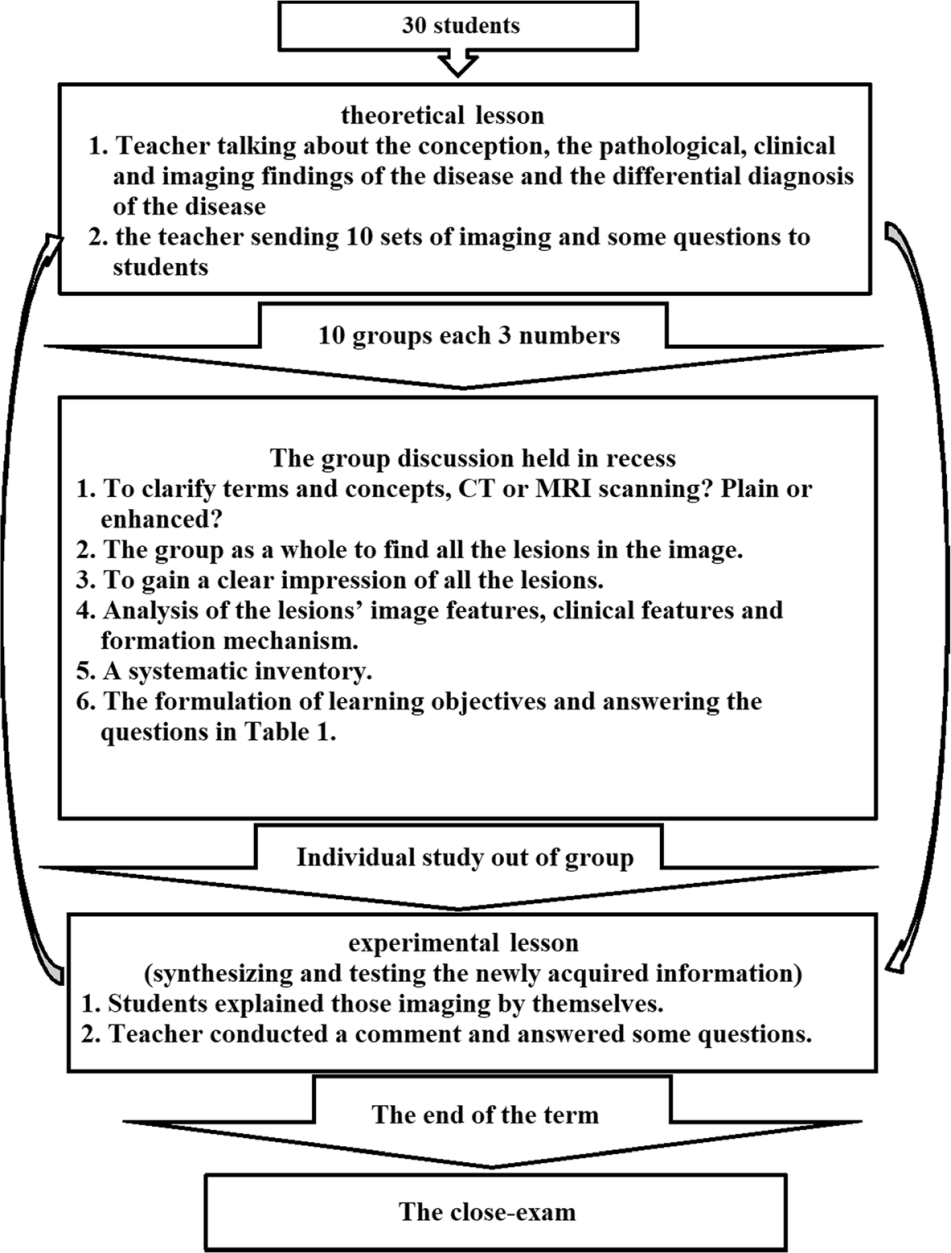
shows the process of integration of the PBL and LBL teaching modes

**Fig. 4:**
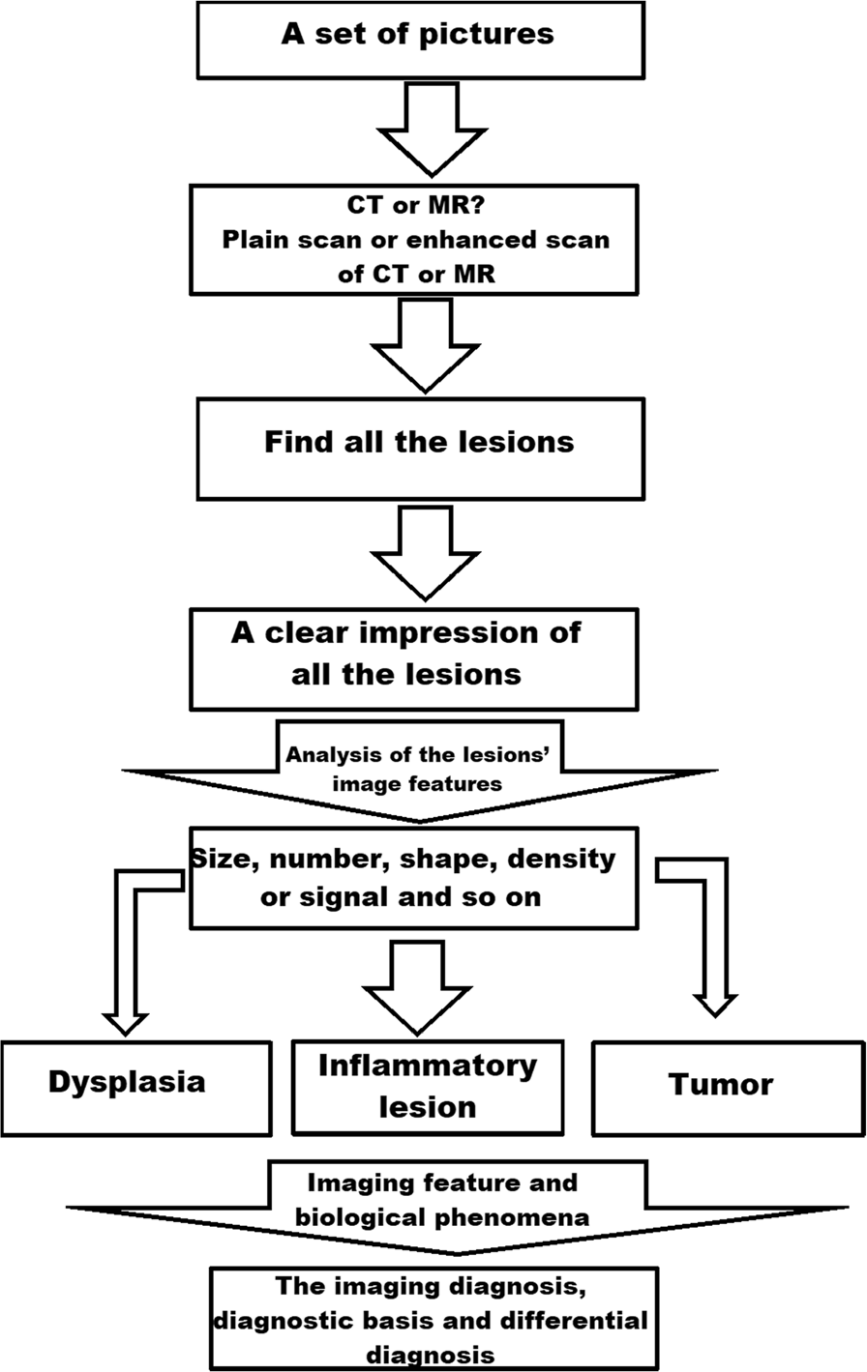
shows the scheme of the systematic inventory

1. For both groups, each module of teaching content included 2 parts (theoretical lessons [approximately 80 min] and experimental lessons, including 10 sets of imaging in each class [approximately 160 min]).
2. The LBL teaching mode is shown in Fig. 2. In the theoretical lessons, teachers talked about the conception, the pathologic, clinical, and imaging findings of the disease, and the differential diagnosis of the disease, while the students listened carefully and took notes. In the experimental lessons, teachers provided imaging pictures. In the initial part of the class, teachers discussed the diagnosis based on the pictures and the imaging findings, including the main points in Table 1. In the latter part of the class, the students evaluated the pictures and asked questions.
3. Integration of the PBL and LBL teaching modes is shown in Fig. 3. First, in the theoretical lessons in which a LBL teaching method was used, the teacher talked about the conception, the pathologic, clinical, and imaging findings of the disease, and the differential diagnosis of the disease, while the students listened carefully and took notes, which assessed prior knowledge. At the end of the course, the teacher sent 10 sets of images and questions (Table 1) to students for discussion in the experimental lessons, which was the start of the PBL teaching method. In addition, the group discussion was held in recess with a teacher present. In this course, the first step was to clarify terms and concepts not understood on initial inspection. For example, which kind of examination does this set of imaging pictures belong to (X-ray, CT, or MRI)? Is it a plain CT or MRI scan, an enhanced CT or MRI scan, or a plain and enhanced CT or MRI? The second step permitted the group as a whole to identify all of the lesions in the image. The third step was to gain a clear impression of all the lesions. The fourth step was an analysis of the imaging features of the lesions, the clinical features, and the mechanism of formation. Most of the time a free association round was held, in which each member was allowed to share thoughts freely before the ideas, knowledge, and supposition were scrutinized, accepted, complemented, or modified. The fifth step of a systematic inventory was made of various explanations of the problem. In the above analysis, several general descriptions of the imaging features and biological phenomena were advanced. The systematic inventory might yield the scheme shown in Fig. 4. The sixth step was the formulation of learning objectives. The answers to the questions elicited in the problem analysis phase are shown in Table 1, and facilitate a more profound knowledge of the processes forming the crux of the problem. Third, the individuals studied outside the group. The group members individually collected information with respect to the learning objectives using teaching materials, library resources, and online information, and they also can consult teachers about aspects of the problem not yet clarified. Finally, the newly acquired information was synthesized and tested. In the experimental lessons, each team of students randomly took one set of images and answered the questions shown in Table 1. Then, the teacher commented on what they had talked about and added what they had not talked about. At the end of the session, the students could ask some questions that they did not understand.
4. Each module of teaching content was discussed by the same teacher and all content was discussed by seven teachers.

**Table 1:** Major learning points of each set of imaging

point 1	What is the imaging diagnosis of these pictures?
point 2	Which imaging signs in these pictures may be used to support your diagnosis?
point 3	Are there any other imaging signs which can also be used to support your diagnosis but not included in these pictures?
point 4	Which diseases should be differentiated with it?

### Data analysis and student survey

At the end of each term, there was a 3-h close-exam covering all of the course materials. The test scores were analysed by means of analysis of variance with a two-phase cross-over design.

At the end of academic year, students completed an anonymous survey to assess their exposure to model experiences. The survey was delivered on the last day of the academic year after the exam. The survey was developed by one of the study authors (Liang C.H.) for the purpose of assessing the curriculum change and piloted during the trial period. Students were asked their opinion of the teaching reform (for definitions, see the survey questions in Table 2). The difference between the two teaching models was compared using a χ2 test with a 2-tailed α of 0.05.

**Table 2:** Themes from teaching reform survey collected from 60 students of imageology specialty at Xinxiang Medical University during 2016–2017 academic years

Themes	No. (percent) of integrated problem- and lecture-based learning teaching mode	No. (percent) of lecture-based learning teaching model	χ^2^	*P*
Which teaching model do you like?	46(76.7%)	14(23.3%)	34.133	0.000
Which teaching model can make your ability of self-study be improved?	53(88.3%)	7(11.7%)	70.533	0.000
Which teaching model can improve your language competence?	55(91.7%)	5(8.3%)	83.333	0.000
Which teaching model can cultivate the team cooperation spirit?	54(90.0%)	6(10.0%)	76.800	0.000
Which teaching model can train your the basic skills?	58(96.7%)	2(3.3%)	104.533	0.000
Which teaching model can cultivate your ability to find, ask, analyze and solve the problems?	57(95.0%)	3(5%)	97.200	0.000
Which teaching model can cultivate active classroom atmosphere?	59(98.3%)	1(1.7%)	112.133	0.000
Which teaching model can expand your personal confidence?	58(96.7%)	2(3.3%)	104.533	0.000
Which teaching model can strengthen teacher-student emotion blend?	54(90.0%)	6(10.0%)	76.800	0.000
Which teaching model can strengthen student-student communication?	54(90.0%)	6(10.0%)	76.800	0.000
Which teaching model can increase pressure on going to class?	58(96.7%)	2(3.3%)	104.533	0.000
Which teaching model, do you think, takes your too much spare time?	35(58.3%)	25(41.7%)	3.333	0.068

## Results

There was a statistically significant difference in the test scores between the integration of the PBL and LBL teaching modes and the LBL teaching mode alone (the score of the former was higher than the latter) and a statistically significant difference in the test scores between the first and second terms (the score of the first term of 60 students was lower than the second term; Table 3).

**Table 3:** Comparative analysis of test scores between different factors

	Test scores($\overline{\upchi}$±s)	*F*	*P*
integrated problem- and lecture-based learning teaching modes	79.24 ± 4.93	247.743	0.000
The lecture-based learning teaching mode	78.25 ± 4.96		
The first term(mean score of 60 students)	78.36 ± 5.15	31.386	0.000
The second term(mean score of 60 students)	79.03 ± 4.88		

Every one of the surveys had a response rate, and the integration of the PBL and LBL teaching modes was well-appraised (Table 2). Forty-six of sixty students (76.7%) like integrated problem- and lecture-based learning teaching mode and 53 of 60 students (88.3%) think that integrated problem- and lecture-based learning teaching mode can make their ability of self-study be improved.

## Discussion

The main findings of this study were as follows: (1) there was a significant increase in the students’ test scores of the integrated PBL and LBL; and (2) the survey data showed that most of the students liked the integration of the PBL and LBL, and the integration of the PBL and LBL did not take much more time than the LBL teaching model. So integration of PBL and LBL into the teaching of imaging diagnosis education is feasible.

Although many people think the LBL teaching mode is not as good as other teaching modes [9–12], different teaching modes have different advantages. If the LBL teaching mode is used properly, the LBL teaching mode can provide maximum benefits. When integrating the PBL and LBL teaching modes, LBL the teaching mode was used in the theoretical lessons of medical imaging so that the teachers could give a systematic state of the etiology, pathophysiology, clinical characteristics, imaging expression, and the course of disease, and this information could be used as prior knowledge and save students hours of searching basic knowledge. The PBL teaching mode was applied in recess and experimental lessons so that the students could actively participate in the course to express their ideas based on prior leaning and experiences obtained from theoretical lessons. Furthermore, students also received feedback from other members of the team. Therefore, students not only receive systematic knowledge, but also actively participate in the course and increase their initiative. The finding of this study that the significant increase in the students’ test scores of the integration of the PBL and LBL support this finding. In contrast, if the LBL teaching mode was used in both theoretical and experimental lessons, students usually just listen, but do not actually participate in the course and do not master what they are told by the teachers. In the current study the students’ test scores of the integrated PBL and LBL teaching modes was higher than the LBL teaching mode.

Survey was used to evaluate the teaching effect. The survey data show that most of the students (76.6%) preferred the integrated PBL and LBL teaching modes and this finding is in agreement with other studies [13, 14]. According to the survey data, the main reason may be as follows: in the integrated PBL and LBL teaching modes, if the students want to answer the questions, they had to search the literature, which can enhance their ability of self-study (88.3%), train their basic skills (96.7%), and cultivate their ability to find, ask, analyse, and solve the problems (95.0%). The students had to talk with each other in the process of team discussion and releasing the answers, which could improve the language competence (91.7%), cultivate their team cooperative spirit (90.0%), strengthen student-student communication (90.0%), and expand personal confidence (96.7%). With respect to releasing the answers, one person on the team answered the question, and other students added new ideas. In the end, the teacher would give a conclusion and post some further explanations, which could cultivate active classroom atmosphere (98.3%) and strengthen the teacher-student emotional bond (90.0%). They had to prepare for the class, which would increase the pressure to attend class (96.7%). The preparation required approximately the same length of time as attending class (*P* = 0.068).

There were several limitations to this study. The sample size of the study was relatively small. In addition, a pure PBL teaching mode was not used in this study. Another important consideration is that the survey was not sufficiently detailed so the time needed to prepare for the experimental course was not recorded.

Further studies should compare the teaching effect of the PBL teaching mode imported into the theoretical and experimental lessons with the integration of PBL and LBL. Further studies could use more detailed message from students undergoing such an experiment.

## Conclusion

Integration of PBL and LBL into the teaching of imaging diagnosis education could achieve a good teaching effect. Not only would students’ test scores improve, but students’ interest in learning would increase, thus improving their ability to self-study, cultivate their team cooperative spirit, strengthen student-student communication, and expand their personal confidence.

## Abbreviations

LBL: Lecture-based learning
PBL: Problem-based learning
